# Detergent-induced quantitatively limited formation of diacyl phosphatidylinositol dimannoside in *Mycobacterium smegmatis*

**DOI:** 10.1016/j.jlr.2024.100533

**Published:** 2024-03-24

**Authors:** Claire E. Kitzmiller, Tan-Yun Cheng, Jacques Prandi, Ian L. Sparks, D. Branch Moody, Yasu S. Morita

**Affiliations:** 1 Department of Microbiology, University of Massachusetts, Amherst, MA, USA; 2 Division of Rheumatology, Immunity and Inflammation, Brigham and Women's Hospital, Harvard Medical School, Boston, MA, USA; 3 Institut de Pharmacologie et de Biologie Structurale, CNRS, UPS, Université de Toulouse, Toulouse, France

**Keywords:** glycolipids, phospholipids, membrane fluidity, metabolism, reverse micelle extraction, stress response, chemical synthesis, biosynthesis

## Abstract

Mycobacterial plasma membrane, together with the peptidoglycan-arabinogalactan cell wall and waxy outer membrane, creates a robust permeability barrier against xenobiotics. The fact that several antituberculosis drugs target plasma membrane-embedded enzymes underscores the importance of the plasma membrane in bacterial physiology and pathogenesis. Nevertheless, its accurate phospholipid composition remains undefined, with conflicting reports on the abundance of phosphatidylinositol mannosides (PIMs), physiologically important glycolipids evolutionarily conserved among mycobacteria and related bacteria. Some studies indicate cardiolipin, phosphatidylethanolamine, and phosphatidylinositol as dominant structural phospholipids. Conversely, some suggest PIMs dominate the plasma membrane. A striking example of the latter is the use of reverse micelle extraction, showing diacyl phosphatidylinositol dimannoside (Ac_2_PIM2) as the most abundant phospholipid in a model organism, *Mycobacterium smegmatis*. Our recent work reveals a rapid response mechanism to membrane-fluidizing stress in mycobacterial plasma membrane: monoacyl phosphatidylinositol dimannoside and hexamannoside (AcPIM2 and AcPIM6) are converted to diacyl forms (Ac_2_PIM2 and Ac_2_PIM6). Given the dynamic nature of PIMs, we aimed to resolve the conflicting data in the literature. We show that unstressed *M. smegmatis* lacks an Ac_2_PIM2-dominated plasma membrane. Ac_2_PIM2 accumulation is induced by experimental conditions involving sodium docusate, a component of the reverse micellar solution. Using chemically synthesized PIMs as standards, we accurately quantified phospholipid ratio in *M. smegmatis* through liquid chromatography-mass spectrometry, revealing that mycobacterial plasma membrane is dominated by cardiolipin, phosphatidylethanolamine, and phosphatidylinositol. PIMs are quantitatively minor but responsive to environmental stresses in *M. smegmatis*. Our study paves the way for accurate modeling of mycobacterial plasma membrane.

## Graphical abstract

**Figure:**
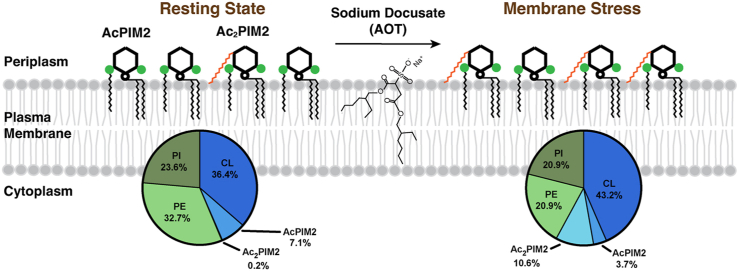

Plasma membrane is the innermost layer of bacterial cell envelope and functions as a semipermeable barrier to protect the cells. Furthermore, bacteria typically do not have membrane-bound subcellular organelles, and therefore plasma membrane must serve as the main platform for reactions mediated by membrane-bound enzymes. The bilayer of phospholipids accounts for the fundamental biophysical properties of the plasma membrane such as lateral fluidity and vertical permeability to create this dynamic milieu. In *Mycobacterium smegmatis*, plasma membrane was proposed to be rich in cardiolipin (CL), phosphatidylethanolamine (PE), and phosphatidylinositol (PI). In an early study using [^32^P]phosphate radiolabeling, LaBelle and Walker estimated that CL and PE account for 31.7% and 28.9% of total phospholipids in *M. smegmatis* (1). PI was not resolved from PI mannosides (PIMs) in their study and accounted for 30.5% in combination. Another study in 2000 by Jackson *et al.* also reported similar values: 37.3% CL, 31.6% PE, 23.4% PI, and 4.5% monoacyl PI dimannoside (AcPIM2) (2). More recently, Angala *et al.* measured steady-state levels of phospholipids in *M. smegmatis* by high performance liquid chromatography-mass spectrometry. While the relative molar abundance of PIMs was higher than early studies, PIMs were still less abundant than PE, CL, and PI (3).

A strikingly different view was proposed in 2014 by Bansal-Mutalik and Nikaido (4). While previous studies used [^32^P]phosphate for phospholipid radiolabeling, they used [^14^C]glucose for universal lipid labeling. Furthermore, they developed a novel reverse micelle extraction to “shave off” the outer membrane lipids before extracting plasma membrane lipids by standard chloroform/methanol-based extraction. This new method revealed striking abundance of a specific PIM molecule, diacyl PI dimannoside (Ac_2_PIM2) in the plasma membrane, and the authors proposed that the inner leaflet of the plasma membrane is nearly entirely made of this glycolipid. Separately, several studies found that PIMs are dominant in the mycobacterial cell envelope, including work by Akamatsu *et al.* in 1966 that found PIMs to make up 40.2% of phospholipids in the whole cell (5). Similarly, Oka *et al.*, in 1968, found that PIMs make up 28%–40% of the cell envelope fractions while PE and CL make up 26%–34% and 27%–43%, respectively (6). However, the studies by Akamatsu and Oka were conducted in *Mycobacterium* species other than *Mycobacterium smegmatis*, namely *M. phlei* and P6 (an unclassified species), respectively. There was no evidence that *M. smegmatis* is PIM-dominant before the study by Bansal-Mutalik and Nikaido. Furthermore, the studies by Akamatsu and Oka were unable to differentiate different forms of PIMs.

PIMs are synthesized in the plasma membrane and can carry up to six mannoses and four fatty acyl chains (Fig. 1A). AcPIM2 and monoacyl PI hexamannoside (AcPIM6) carry two and six mannoses respectively, with one of the mannoses modified by fatty acylation. These tri-acylated forms were previously considered major PIM species, and Ac_2_PIM2, a tetra-acylated lipid, was thought to be quantitatively minor prior to the proposal by Bansal-Mutalik and Nikaido. For example, metabolic PIM labeling using [^3^H]inositol or [^3^H]mannose, chemical lipid staining, and mass spectrometry (MS) consistently revealed AcPIM2 and AcPIM6 as major species compared with Ac_2_PIM2 and diacyl PI hexamannoside (Ac_2_PIM6) in both *M. smegmatis* and *Mycobacterium tuberculosis* (7, 8, 9, 10, 11, 12, 13).

**Fig. 1:**
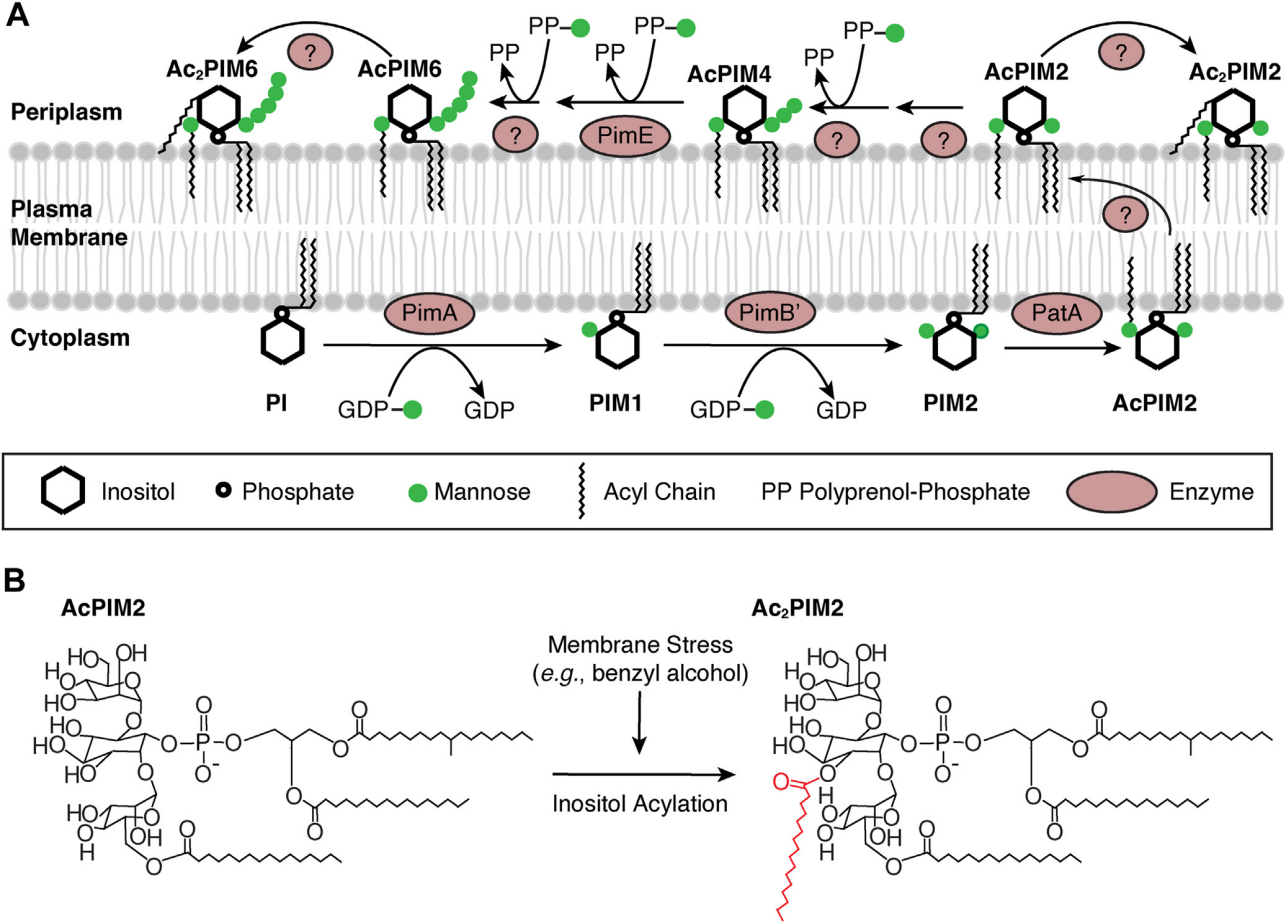
PIM biosynthesis and inositol acylation. A: Biosynthesis of AcPIM4, AcPIM5, and AcPIM6 are dependent on polyprenol-phosphate-mannose (PPM) as a mannose donor (17), suggesting that these steps take place in the periplasmic leaflet. For the biosynthetic steps between AcPIM2 and AcPIM6, PimE is the only known mannosyltransferase (16). Both AcPIM2 and AcPIM6 are readily available for inositol acylation reaction (19), implying that they are on the same leaflet. Based on these observations, we speculate that AcPIM2 flops from the cytoplasmic leaflet to the periplasmic leaflet (see main text for detailed discussion). B: Inositol acylation induced by membrane fluidization stress (19). The fatty acid (shown in red) is esterified at the 3-OH of the *myo*-inositol ring (23).

PIM biosynthesis starts with PimA (MSMEG_2935) mannosyltransferase, which transfers a mannose from GDP-mannose to the 2-position of the *myo*-inositol ring of PI, forming PIM1 (11). PimB’ (MSMEG_4253) then transfers another mannose to the 6-position of the *myo*-inositol ring using GDP-mannose as a mannose donor, forming PIM2 (14, 15). PatA (MSEMG_2934) acyltransferase transfers a fatty acid to the 6-position of the mannose residue attached to the 2-position of the *myo*-inositol ring (12), resulting in AcPIM2. Four more mannoses are added to AcPIM2 to make AcPIM6, but the enzymes are unknown, except the mannosyltransferase PimE (MSEMG_5136) that adds the fifth mannose (16) (Fig. 1A). Some of these reactions have been demonstrated in a cell-free PIM biosynthesis system using GDP-[^3^H]mannose as a mannose donor, and Ac_2_PIM2 was again a minor radiolabeled species compared with AcPIM2 (17). Hence, the recent finding that Ac_2_PIM2 is the most abundant lipid in the plasma membrane among other phospholipids was surprising.

As bacteria belonging to the Gram-positive lineage, mycobacteria are unusual in having an outer membrane. Compositional analysis of the outer membrane versus the inner membrane has been difficult in mycobacteria due to the inability to effectively separate the outer membrane from the cell wall and plasma membrane. Therefore, the application of the reverse micelle extraction method developed by Bansal-Mutalik to the analysis of diderm membrane structures in mycobacteria and corynebacteria was groundbreaking (4, 18). The unexpected abundance of Ac_2_PIM2 extracted from the plasma membrane after the extraction of outer membrane lipids by reverse micellar solution (RMS) was explained to be due to the “loosening” of the cell envelope by RMS treatment, resulting in more efficient extraction of Ac_2_PIM2 from the inner membrane. However, we recently showed that both AcPIM2 and AcPIM6 undergo rapid inositol acylation to become Ac_2_PIM2 and Ac_2_PIM6 in response to membrane stress (Fig. 1B). The entire population of monoacyl PIMs can be converted stoichiometrically to diacyl PIMs in 10–15 min not only by membrane fluidizing chemicals such as benzyl alcohol but also by heat shock at 55°C without any chemical treatment (19). In all cases, these diacyl PIMs are readily extracted by standard chloroform/methanol-based extractions without a prior RMS treatment. We wondered if the dominance of Ac_2_PIM2 after RMS treatment was a stress response of *M. smegmatis* cells rather than loosening of the cell envelope. Here we show that *M. smegmatis* remains viable during the RMS treatment, and sodium docusate, the detergent component of RMS, induces the accumulation of Ac_2_PIM2. These results emphasize the low abundance of AcPIM2 in the inner membrane and the dynamic nature of phospholipid remodeling.

## Materials and methods

### Cell culture

*Mycobacterium smegmatis* mc^2^155 (20) was grown in Middlebrook 7H9 (BD Difco) supplemented with 15 mM NaCl, 0.2% (w/v) glucose, 0.2% (v/v) glycerol, and 0.05% (v/v) Tween-80 (dextrose-sodium chloride supplement). Where indicated, Middlebrook 7H9 was supplemented with 15 mM NaCl, 0.2% (w/v) glucose, 0.5% (w/v) bovine serum albumin, and 3 μg/ml catalase (albumin-dextrose-catalase [ADC] supplement) without the addition of Tween-80. All cultures were grown shaking at 120 rpm at 37°C. Colony forming units (CFU) were determined by spotting 5 μl of serially diluted cell cultures on Middlebrook 7H10 agar supplemented with 15 mM NaCl, 0.5% (v/v) glycerol, and 0.2% (w/v) glucose.

### Rapid heat-killing

To kill *M. smegmatis* cells rapidly without inducing heat-shock responses, 100 ml of cells in a 500-ml flask were microwaved at 1,250 W for 30 s three times with brief mixing in between.

### Lipid extraction by RMS

For outer membrane lipid extraction, 40 *A*_600_ units of cells were centrifuged at 3,220 x *g* for 10 min, and the pellet was resuspended in 10 volumes of RMS (10 mM sodium docusate in heptane). After extraction at either room temperature or −20°C for up to 22 h, cells were pelleted by centrifugation, and the supernatant was collected.

### Lipid extraction by chloroform/methanol/water

For inner membrane lipid extraction, RMS-delipidated cell pellets were centrifuged at 3,220 x *g* for 10 min, and the pellet was resuspended in 20 volumes (relative to the wet pellet weight) of chloroform/methanol (2:1, v/v) and briefly vortexed. Following a 1.5-h room temperature incubation, the suspension was spun down at 3,220 x *g* for 2 min, and the supernatant was collected. This process was repeated with 10 volumes of chloroform/methanol (2:1, v/v), and then 10 volumes of chloroform/methanol/water (CMW) (1:2:0.8, v/v/v) against the same pellet. The combined extracts were dried under nitrogen stream, and lipids were further purified by 1-butanol/water (2:1, v/v) phase partitioning. The butanol phase was dried and resuspended at 1 mg wet pellet equivalent per μL of water-saturated butanol. For total lipid extraction (without RMS treatment), the same procedure was followed using the wet pellet of 40 *A*_600_ units of cells as a starting material.

### High-performance thin-layer chromatography

For the analysis of PIMs and other phospholipids, purified lipids were developed on an aluminum-backed high-performance thin-layer chromatography (HPTLC) plate (silica gel 60, EMD Merck) sequentially in hexane and then chloroform/methanol/13 M ammonia/1 M ammonium acetate/water (180:140:9:9:23, v/v/v/v/v). PIMs were visualized by orcinol staining. Phospholipids were detected by Molybdenum Blue staining (Millipore-Sigma). Lipid bands on HPTLC plates were scanned for densitometric measurements as described previously (21). Dipalmitoyl phosphatidylglycerol (DPPG) was purchased from Millipore-Sigma. Since lipids were extracted using calculated volumes of organic solvents based on the wet cell pellet weight, equal amounts of total lipids derived from equal number of cells were loaded for all HPTLC analysis. This standardized protocol achieved reproducible and consistent lipid loading as seen in the biological quadruplicate sample analysis (see below).

**Fig. 2:**
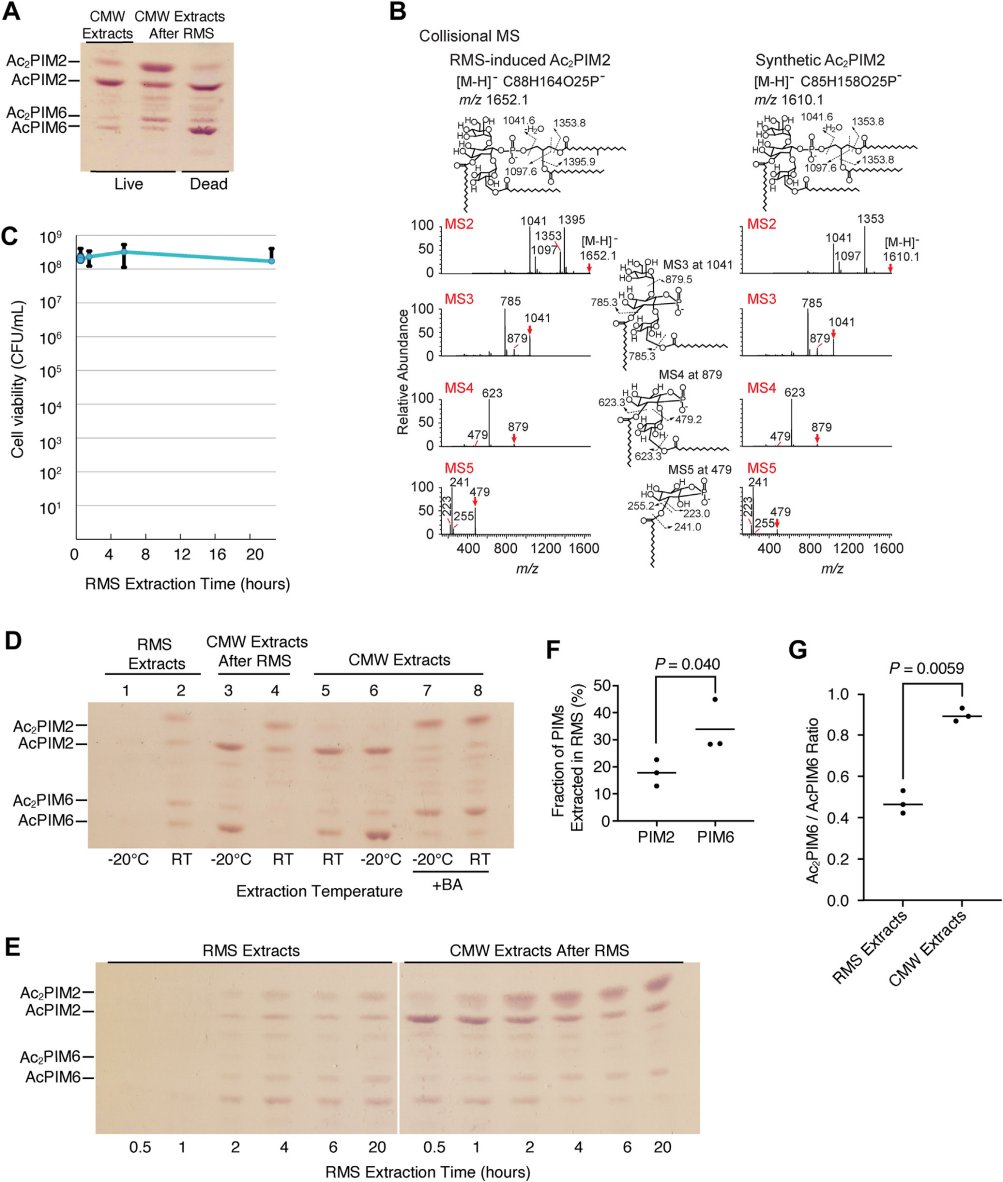
Diacyl PIMs accumulate in live *M. smegmatis* in response to RMS treatment. A: HPTLC analysis of PIMs extracted into RMS and CMW from live and heat-killed cells, following the published extraction protocol (4). PIMs were chromatographed using a solvent containing chloroform, methanol, 13 M ammonia, 1 M ammonium acetate, and water (180:140:9:9:23, v/v/v/v/v) and visualized by orcinol staining. A region of the HPTLC plate corresponding to Rf of 0.30–0.56 is shown. B: HPTLC-purified Ac_2_PIM2 induced by RMS treatment was analyzed by linear ion trap multistage collisional MS compared to the synthetic molecule Ac_2_PIM2. The location of the fatty acyl chain on the inositol was inferred from the literature (23) but cannot be determined in the current experiment. C: *M. smegmatis* cells remain viable during RMS treatment. Log phase cells were treated with RMS, resuspended in Middlebrook 7H9 media at indicated time points, serially diluted, and plated onto Middlebrook 7H10 agar. D: PIM inositol acylation depends on the temperature during RMS treatment. PIMs were extracted by RMS then CMW or directly by CMW without RMS at the indicated temperature and analyzed by HPTLC as in panel A (Rf = 0.29–0.58). Cells for lanes 7 & 8 were first treated with 100 mM benzyl alcohol (BA) for 1 h at room temperature before CMW extraction at the indicated temperature. E: Time course of PIM inositol acylation during the RMS treatment. Lipids were extracted from log phase cells into RMS for the indicated amounts of time and remaining lipids were extracted into CMW. PIMs were analyzed by HPTLC as in panel A (Rf = 0.27–0.61). Two parts separated by a white line are cropped from the same HPTLC plate. F: PIM6 species are more prone to RMS extraction than PIM2 species. Log phase cells were treated with RMS for 4 h, followed by CMW extraction of remaining lipids. Combined band intensities of PIM2 species (AcPIM2 and Ac_2_PIM2) were measured by densitometry and compared with those of PIM6 species (AcPIM6 and Ac_2_PIM6). *P* value of biological triplicates was determined by the Student’s *t* test. G: Ac_2_PIM6 is more enriched in the CMW extract. From the same experiment described in panel F, the ratio of Ac_2_PIM6 to AcPIM6 was calculated from the densitometric measurements. *P* value was determined by the Student’s *t* test. RT, room temperature.

**Fig. 3:**
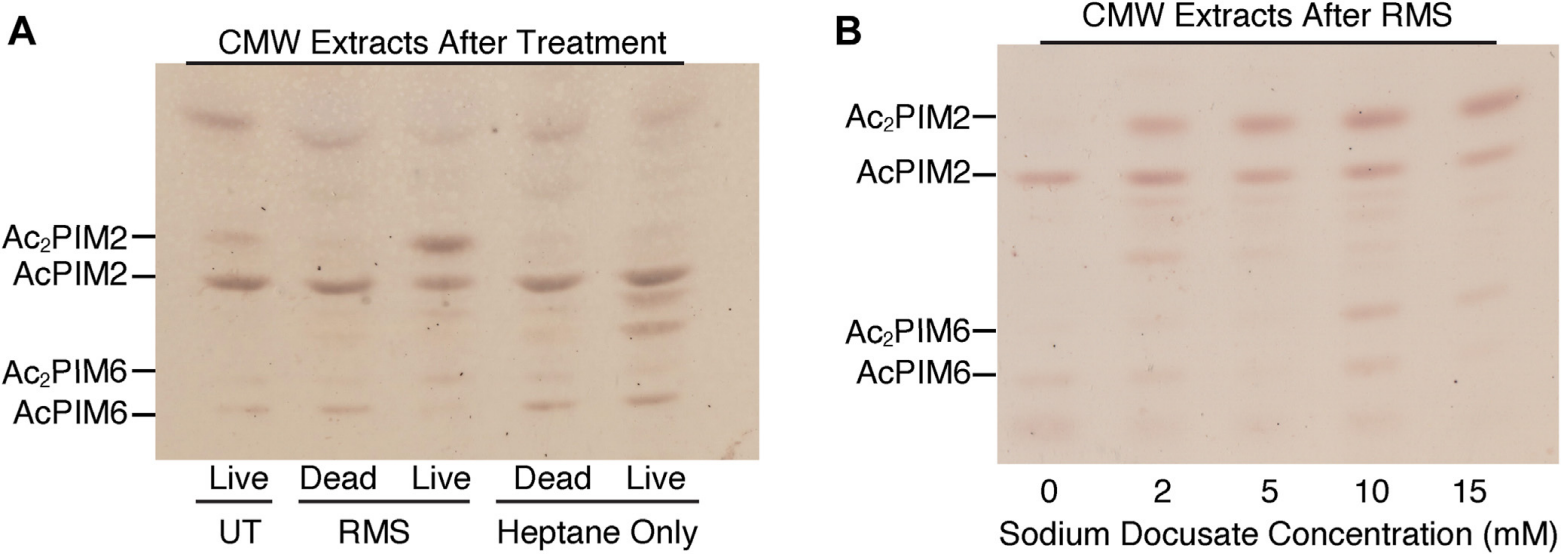
Heptane alone does not induce inositol acylation. A: HPTLC analysis of PIMs extracted into CMW from live and heat-killed cells. Cells were either untreated (UT, lane 1), treated with RMS (lanes 2&3), or treated with heptane (lanes 4&5) before CMW extraction. PIMs were analyzed as in Fig. 2A (Rf = 0.28–0.65). B: Lipids were extracted into RMS with varying concentrations of sodium docusate, and the remaining lipids were extracted into CMW. The HPTLC analysis (as in Fig. 2A) of the CMW extracts is shown (Rf = 0.24–0.63).

**Fig. 4:**
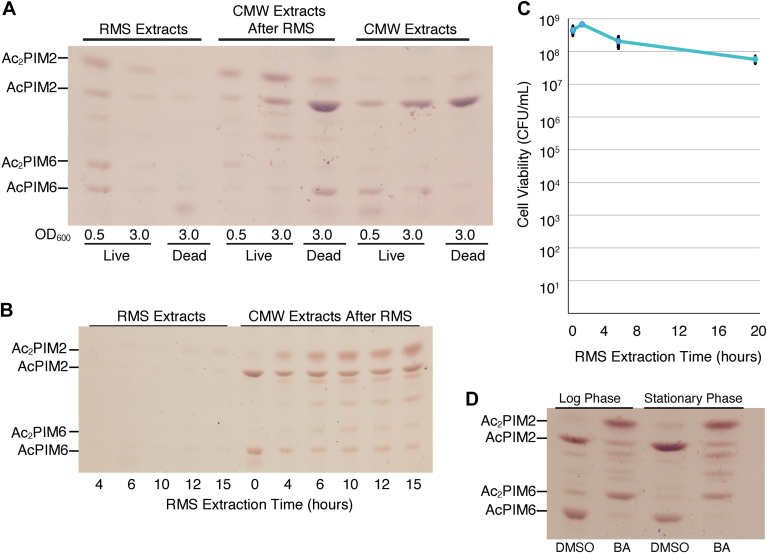
PIM inositol acylation in different growth stages. A: HPTLC analysis of PIMs extracted into RMS, CMW after RMS treatment, and CMW without RMS treatment from live and heat-killed cells at an *A*_600_ of 0.5 or 3.0. PIMs were analyzed as in Fig. 2A (Rf = 0.25–0.64). B: Lipids were extracted from early stationary phase cells into RMS for the indicated amounts of time, and remaining lipids were extracted into CMW. PIMs were analyzed as in Fig. 2A (Rf = 0.19–0.62). C: Early stationary phase cells were treated with RMS for indicated time points, resuspended into Middlebrook 7H9 media, serially diluted and plated onto Middlebrook 7H10 agar for CFU counting. D: Log phase and early stationary phase cells were treated with DMSO (vehicle control) or 100 mM benzyl alcohol (BA) for 1 h before CMW lipid extraction. PIMs were analyzed as in Fig. 2A (Rf = 0.19–0.50).

**Fig. 5:**
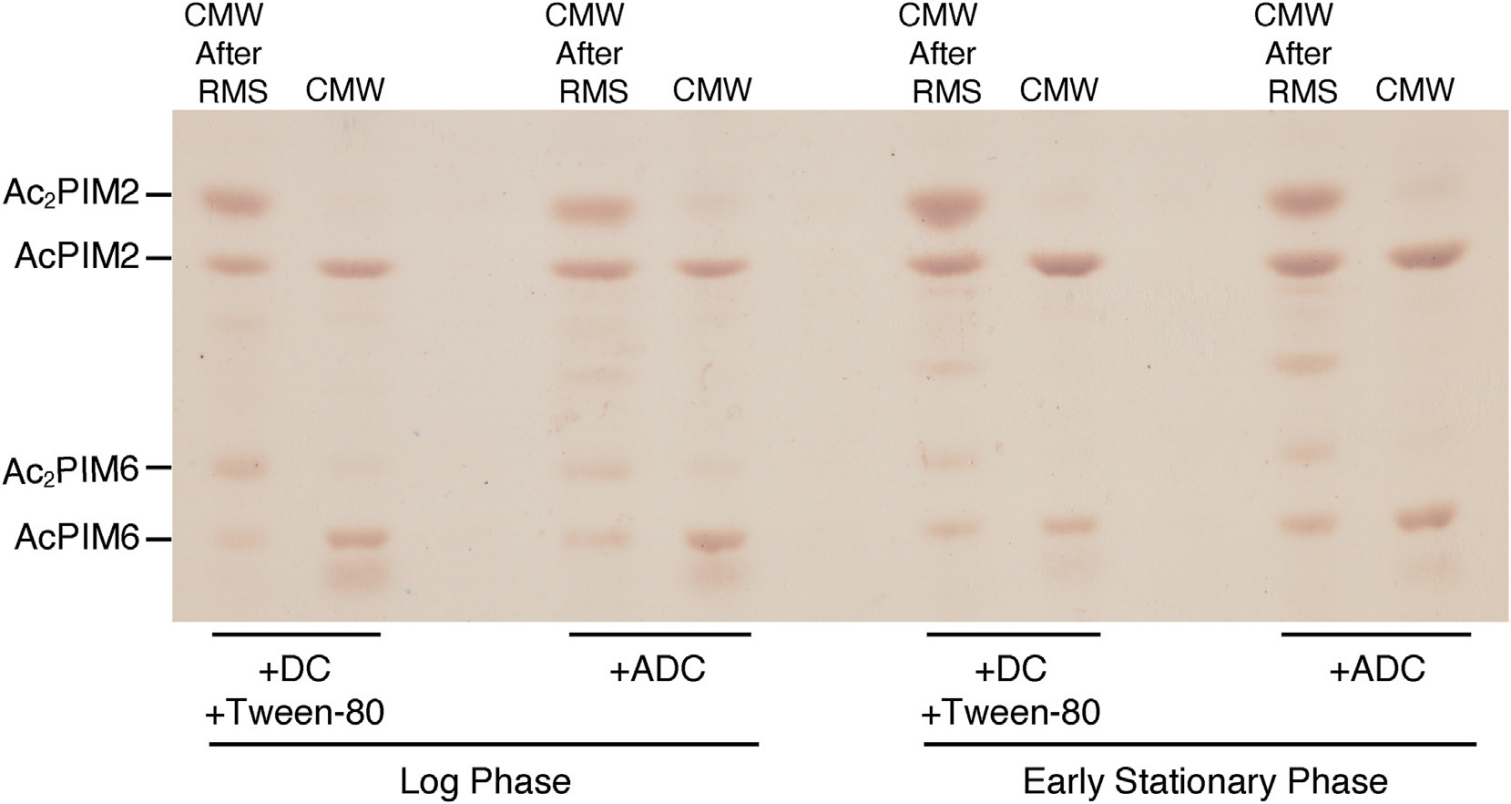
Effect of medium components on PIM inositol acylation. Cells were grown to log or early stationary phase in Middlebrook 7H9 supplemented with ADC or DC and Tween-80. PIMs were extracted into CMW after the RMS treatment or directly into CMW. PIMs were analyzed as in Fig. 2A (Rf = 0.26–0.66). DC, dextrose-sodium chloride.

**Fig. 6:**
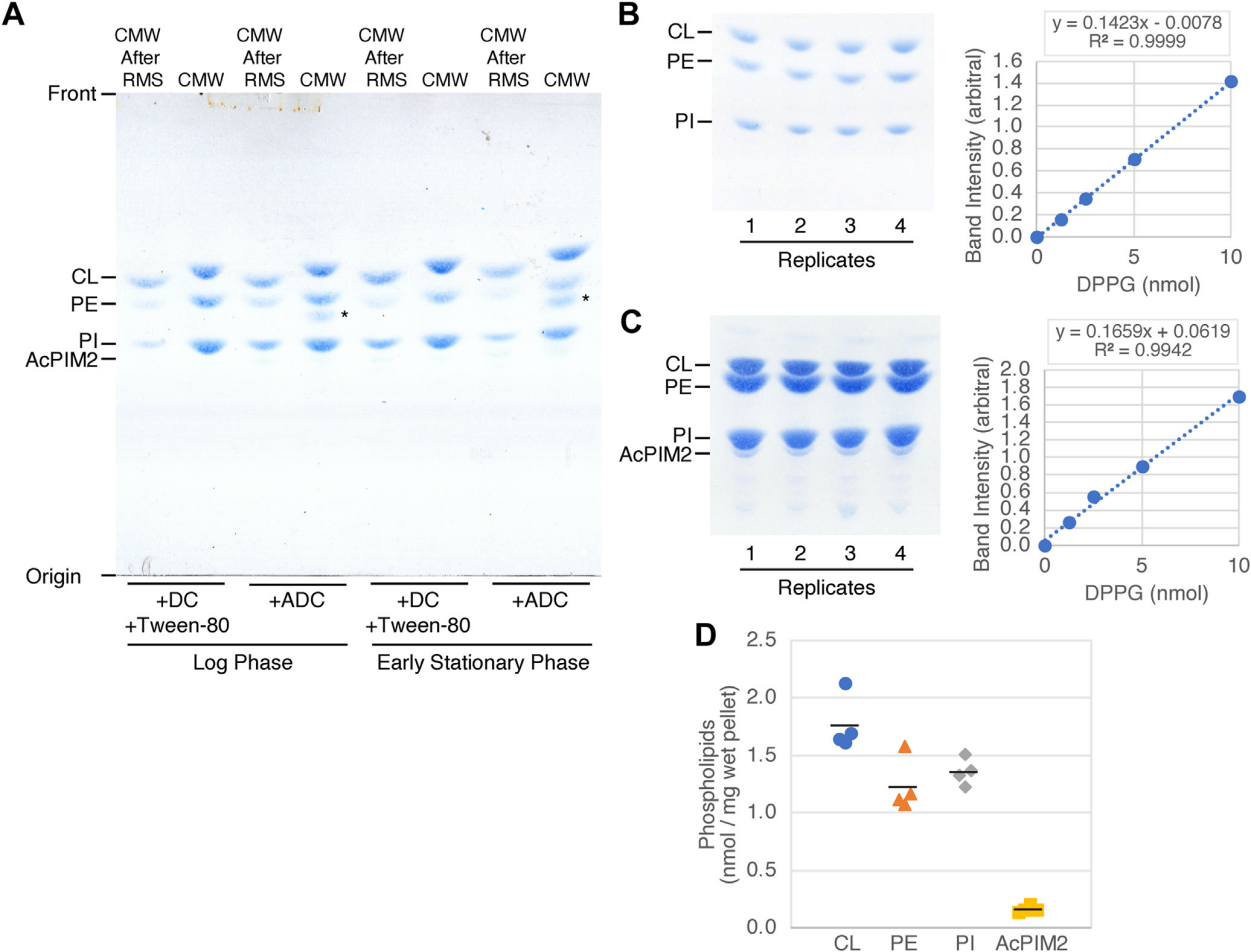
PIMs are not as abundant as other phospholipids. A: Lipid extracts from the same experiment shown in Fig. 5 were analyzed by HPTLC as in Fig. 2A and stained for phospholipids by Molybdenum Blue. ∗, unknown phospholipid. B: CMW extracts from 1.5 mg wet pellet of log phase cells grown in the standard growth medium were analyzed in biological quadruplicate alongside with DPPG standards. Lipid analysis was as described in panel A. Band intensities of CL, PE, and PI, measured by FIJI as previously described (21), were in the ranges of 0.336–0.445, 0.222–0.329, and 0.254–0.315 (arbitral density unit), which were all within the linear range of the DPPG standard curve. C: Lipids from 12 mg wet cell pellet were analyzed to visualize AcPIM2. Band intensities of AcPIM2 were 0.328–0.477. D: Phospholipid abundance in log-phage *M. smegmatis* cells shown in nmol per milligram wet cell pellet.

### Synthetic lipid standards

PI (C34:1, Cat # 850142), CL (C18:1, Cat # 710335), and PE (C34:1, Cat # 850757) were purchased from Avanti Polar Lipids. PIM2 and Ac_2_PIM2 were synthesized and provided by Dr Jacques Prandi, at the University of Toulouse. Individual standard was prepared as a 100 μM stock solution followed by a serial dilution and then measured by MS to generate a standard curve (chromatogram area vs. concentration).

### Mass spectrometry

For multistage collision-induced dissociation tandem MS analysis, the synthetic Ac_2_PIM2 or HPTLC-purified Ac_2_PIM2 was dissolved in methanol (50 μM) and loaded onto a nanospray glass tip and then analyzed by negative-mode electrospray-ionization MS (LXQ ion trap spectrometer, Thermo Scientific). The spray voltage and capillary temperature were 0.7 kV and 200°C, respectively. Collision energy was set to 20%–35% of maximum, and product ions were trapped with a *q* value of 0.25.

For quantitative HPLC-quadrupole-time-of-flight (Q-TOF)-MS analysis, the bacterial lipids were normalized to 0.5 mg/ml, and 10 μl were injected onto a reversed phase HPLC system (Agilent Poroshell EC-C18 column, 1.9-μm, 3 × 50 mm with a 2.7- μm, 3 × 5 mm guard column) coupled to an Agilent 6546 Accurate-Mass Q-TOF mass spectrometer with HPLC 1260 series. The flow rate was set to 0.15 ml/min, and the gradient conditions were set based on the published method (22). Briefly, the mobile phases (A) 2 mM ammonium formate in methanol/water (95:5, v/v) and (B) 3 mM ammonium formate in 1-propanol/cyclohexane/water (90:10:0.1, v/v/v) were prepared for a 20-min run, and gradients were following: 0–4 min of 100% A, 4–10 min from 100% A to 100% B, 10–15 min of 100% B, 15–16 min from 100% B to 100% A, and 16–20 min of 100% A. The negative ion-mode data were analyzed using the Agilent MassHunter software.

## Results

### Inositol acylation induced by RMS in live metabolically active *M. smegmatis*

We first tested if diacyl PIM species are synthesized after RMS treatment in live cells. We treated live cells sequentially with RMS and CMW at room temperature and analyzed the CMW-extracted lipids by HPTLC. As a negative control, we treated rapidly heat-killed cells (19). For total lipid extracts, we extracted lipids directly from live cells using CMW (Fig. 2A, lane 1). As observed before (4), diacyl PIM species assigned as Ac_2_PIM2 and Ac_2_PIM6 accumulated in CMW-extracted lipids after RMS treatment compared with the total lipid extracts (Fig. 2A, lane 2). To formally confirm the identity of accumulated Ac_2_PIM2 and establish the location of the fourth acyl chain by MS, we purified Ac_2_PIM2 species from RMS-treated cells and analyzed them by nanoelectrospray multistage collision-induced dissociation tandem MS. The multistage MS/MS method offers isolation of precursor ions of stable intermediates during the collision process. We collided the most abundant Ac_2_PIM2 species (3 C16:0/1 C19:0 acyl chains, [M-H]^-^ m/z 1652.1) from the bacterial sample and compared it to the known structure of the synthetic Ac_2_PIM2 (4 C16:0 acyl chains, [M-H]^-^ m/z 1610.1). By losing the diacylglycerol fragment, both molecules yielded the *m/z* 1041 intermediate in MS2 and showed fingerprint patterns from MS3 to MS5 indicating strong similarity of two molecules. Importantly, the ion at *m/z* 479, consistent with the structure of inositol monophosphate carrying a palmitate, was isolated from MS4 and further fragmented to yield the expected inositol head group, of *m/z* 241 and m/z 223 which represents loss of water, as well as palmitate ion, m/z 255, which further supports the structure, indicating that the molecule has an additional fatty acid attached to the inositol moiety (Fig. 2B). While our MS analyses cannot determine the linkage of the acyl chain on the inositol, we posit that the 3-OH of the *myo*-inositol ring is esterified by the acyl chain, based on a published study (23).

Interestingly, there was no accumulation of diacyl PIM species in the CMW extract of heat-killed cells that had been treated with RMS (Fig. 2A, lane 3). This observation implied that the accumulation of diacyl PIM species is a biological response of stressed *M. smegmatis*. To confirm that *M. smegmatis* cells remain viable during RMS treatment, we measured CFU at various time points during RMS extractions. We found no significant decrease in cell viability over the course of 22-h extraction (Fig. 2C), supporting the notion that diacyl PIM accumulation is a response of live cells. If robust accumulation of diacyl PIMs is a consequence of biological reaction at room temperature, we reasoned that the accumulation of diacyl PIMs may be prevented by extracting lipids at −20°C, a temperature that is likely too low to support metabolic reactions. We extracted lipids sequentially using RMS and CMW at −20°C and compared with room temperature extraction (Fig. 2D). At room temperature, some PIMs were extracted by RMS, suggesting that some PIMs may be surface-exposed (Fig. 2D, lane 2). At −20°C, there were no PIMs extracted by RMS, suggesting that RMS extraction is not efficient at this temperature (Fig. 2D, lane 1). The CMW extract after RMS treatment was enriched with diacyl PIMs when the cells were pretreated with RMS at room temperature (Fig. 2D, lane 4). In contrast, when the cells were pretreated with RMS at −20°C, there was no accumulation of diacyl PIM species (Fig. 2D, lane 3), consistent with the possibility that diacyl PIM accumulation is a live cell response of the mesophilic organism. To test that the lack of extraction of diacyl PIMs is not due to an inefficient extraction of diacyl PIMs by CMW at the low temperature, we first treated cells with benzyl alcohol to induce the accumulation of diacyl PIMs and then extracted lipids by CMW at −20°C. The CMW extraction efficiencies of PIMs at room temperature and −20°C were no different, and both monoacyl PIMs (AcPIM2 and AcPIM6, Fig. 2D, lanes 5 and 6) and diacyl PIMs (Ac_2_PIM2 and Ac_2_PIM6, Fig. 2D, lanes 7 and 8) were extracted efficiently at the low temperature. We also examined the time course of diacyl PIM accumulation (Fig. 2E). Consistent with the results shown in Fig. 2D, some PIMs were extracted by the room temperature RMS treatment, but the majority were extracted by the subsequent CMW extraction (Fig. 2E). Furthermore, the extraction of Ac_2_PIM2 and Ac_2_PIM6 in CMW increased gradually over 4 h of RMS treatment (Fig. 2E), suggesting a relatively slow response of inositol acylation compared with fast reaction to benzyl alcohol as reported previously (19). Notably, PIM6 species were more extractable by RMS than PIM2 species (Fig. 2F): in a triplicate experiment, based on densitometric measurements, 28%–45% of total PIM6 species were extracted by 4-h treatment with RMS while only 12%–23% of total PIM2 species were RMS-extractable. Furthermore, within PIM6 species, Ac_2_PIM6 was not extracted by RMS as efficiently as AcPIM6 (Fig. 2G), implying that Ac_2_PIM6, which was newly synthesized in response to the RMS treatment, was not readily available for surface extraction by RMS.

### Inositol acylation induced by sodium docusate

RMS is 10 mM solution of sodium docusate (synonyms, Aerosol OT, or sulfosuccinic acid 1,4-bis(2-ethylhexyl) ester sodium salt) in heptane. Sodium docusate is an anionic detergent with a wide medical use particularly to treat constipation (24, 25). Since heptane is not miscible with water, we suspected that sodium docusate may be the culprit of the PIM inositol acylation response. To test, live and dead cells were treated with heptane alone alongside the standard RMS solution. Heptane alone did not induce PIM acylation, indicating that the accumulation of diacyl PIMs is a response to sodium docusate (Fig. 3A). Additionally, we tested the effect of different concentrations of sodium docusate on PIM inositol acylation during RMS extraction, ranging from 2 to 15 mM. There was robust inositol acylation of AcPIM2 at 5–10 mM sodium docusate while acylation of AcPIM6 required 10 mM or higher, possibly suggesting a substrate preference of inositol acylation at lower sodium docusate concentration (Fig. 3B).

### Effects of growth phase and media composition on PIM inositol acylation

In the previous work (4), Bansal-Mutalik and Nikaido extracted lipids from cells in early stationary growth phase (*A*_600_ = 3.0) and noted that the lipid profile described in their study may be specific to this particular growth phase. We therefore compared PIM abundance in the inner and outer membranes during logarithmic growth phase and early stationary growth phase. We first compared PIM species at an *A*_600_ of 0.5 and 3.0 after overnight RMS extraction and observed an accumulation of diacyl PIMs at both growth phases (Fig. 4A). We then sought to determine the time required for the accumulation of diacyl PIMs during RMS extraction of the stationary growth phase. Early stationary growth phase cells accumulated a minor amount of diacyl PIMs after 4 h of RMS treatment. It took 6–10 h of RMS treatment for diacyl PIMs to accumulate to levels seen after 4 h of RMS treatment with log phase cells (Fig. 4B, compared with Fig. 2E). To confirm that *M. smegmatis* remains viable during RMS treatment at early stationary phase, we measured CFU at various time points during extraction. We found slight reduction in cell viability for the first 8 h and about one log reduction after 20 h of exposure to RMS (Fig. 4C). To confirm that stationary phase cells can rapidly switch their PIMs, we treated cells with benzyl alcohol for 1 h. Dimethyl sulfoxide was used as a vehicle control. We found that cells at early stationary phase were able to acylate PIM inositol within 1 h of growth arrest by the benzyl alcohol treatment (Fig. 4D). The fast response of early stationary phase cells, which is similar to that of log phase cells, suggests that cells at this growth phase are capable of rapidly altering their PIM composition in response to growth-arresting membrane fluidization although their response to RMS solution was somewhat slower.

In the previous work, *M. smegmatis* was grown in Middlebrook 7H9 broth, supplemented with ADC enrichment without the addition of surfactants such as Tween-80. Our standard growth medium for *M. smegmatis* is Middlebrook 7H9 broth, supplemented with dextrose-sodium chloride enrichment and 0.05% Tween-80. To examine whether the growth medium impacts the PIM profile, cells were grown in these two media to log phase or early stationary phase and lipids were extracted by CMW with or without prior RMS treatment. There were no obvious differences in PIM composition of cells at either log or early stationary phase when grown in either medium, except that there was slightly more accumulation of PIM intermediates migrating between AcPIM2 and AcPIM6 when the ADC supplement was used (Fig. 5).

### Minor abundance of PIMs relative to other major phospholipids

Finally, we tested the abundance of PIM species. We first used Molybdenum Blue staining of HPTLC plates as a readout of phospholipid abundance. Molybdenum Blue spray reagent contains molybdenum (VI) oxide and sulfuric acid. Mo (VI) and acid act together to hydrolyze phosphate-containing organic molecules (26, 27, 28, 29). Labile phosphodiester bonds of PIMs and other phospholipids are readily hydrolyzed by this reaction, and released inorganic phosphates then react with Mo (VI) to form intensely colored phosphomolybdenum blue pigments (30). As shown in Fig. 6A, major phospholipid species are CL, PE, and PI regardless of the growth condition. We have not characterized the additional phospholipid species accumulating in cells grown in Middlebrook 7H9 supplemented with ADC (indicated by asterisks). Importantly, AcPIM2, which is the most abundant PIM species based on orcinol staining, is barely detectable by Molybdenum Blue staining, suggesting the quantitatively minor nature of PIM species. These observations are consistent with our previous results (16, 31) as well as other reports described in the Introduction. To accurately quantify phospholipid levels, lipids were extracted from log-phase cells cultivated under standard growth conditions, with four independent biological replicates. The resulting lipid extracts were separated using HPTLC and stained with Molybdenum Blue. DPPG standards were used for reference alongside the sample extracts. The densitometric analysis revealed a linear relationship of DPPG staining intensities and the amounts of DPPG spotted on the HPTLC plate, within the range of 1.25–10 nmol (Fig. 6B, C). For the quantification of CL, PE, and PI, the lipid extracts from 1.5 mg of wet cell pellet were analyzed in one HPTLC run (Fig. 6B). However, the analysis failed to detect AcPIM2 at this loading. With an 8-fold increase in lipid loading for Fig. 6C, AcPIM2 was detected migrating slightly slower than PI. Its band intensities were within the linear range of the DPPG standard curve. The derived phospholipid quantities per milligram of wet cell pellet, determined from the four biological replicates, are summarized in Fig. 6D. On average, CL, PE, and PI constituted 1.77, 1.23, and 1.36 nmol, respectively, while AcPIM2 accounted for 0.16 nmol. Thus, CL, PE, PI, and AcPIM2 collectively represented 39%, 27%, 30%, and 4% of the total major phospholipid content, respectively. Based on orcinol staining, other PIM species, including Ac_2_PIM2, are even less abundant than AcPIM2.

**Fig. 7:**
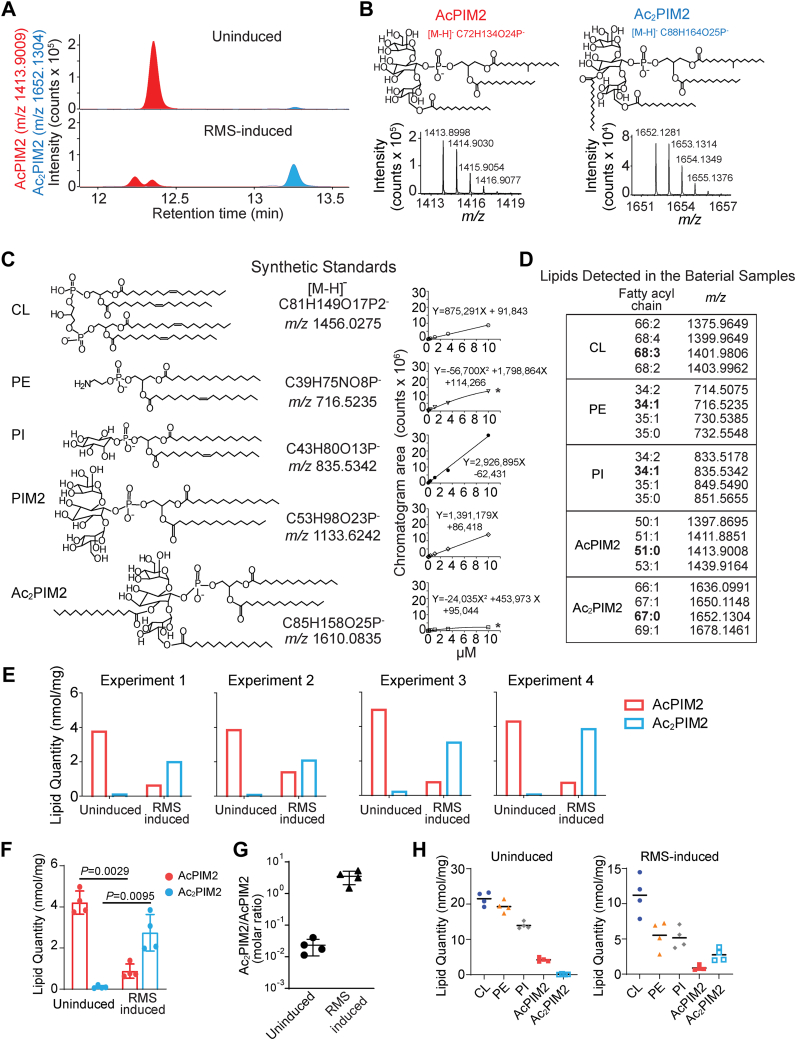
Quantification of AcPIM2 and Ac_2_PIM2 by HPLC-MS. A: Lipid extracts from wet pellets of log phase cells grown in the standard growth medium were analyzed by negative-mode, reversed phase HPLC-Q-TOF-MS in biological quadruplicates. The ion chromatograms of the most abundant species in AcPIM2 (*m/z* 1413.8998) and Ac_2_PIM2 (*m/z* 1652.1281) lipid classes were detected in uninduced and RMS-induced conditions, respectively. A representative chromatogram from one replicate is shown. B: The mass spectrum of the most abundant AcPIM2 and Ac_2_PIM2 shown in panel A with structures based on the literature. C: A series of known concentrations of synthetic compounds, CL, PE, PI, PIM2, and Ac_2_PIM2 were analyzed by HPLC-MS to generate standard curves using linear or nonlinear equations for curve fitting as indicated. ∗the nonlinear fitting results were verified by the linear fitting results with logarithmic scale conversion of both x- and y-axis shown in Supplemental Fig. S1. D: Four major lipid species in each phospholipid class were listed, and the most abundant species was indicated in bold. E: The concentration of AcPIM2 or Ac_2_PIM2 in each condition was converted from the chromatogram area shown in (A) to nmol/mg lipid based on the external standard curve fitting. The quantities of AcPIM2 were estimated by averaging the amounts calculated using both PIM2 and Ac_2_PIM2 standard curve fitting. F: Four experiments in panel E were combined for the statistical analysis using the paired two-tailed Student's *t* test. G: The comparison of Ac_2_PIM2 to AcPIM2 molar ratio in two cellular conditions calculated from panel E. H: The phospholipid abundance was estimated in nmol per mg lipid using all lipid species listed in panel D, based on external standard curve fitting.

PI and Ac_2_PIM2 migrate closely on HPTLC and are difficult to separate. Since Ac_2_PIM2 is present at levels much less than AcPIM2 under standard RMS-untreated conditions, its contribution to the quantification of PI is likely to be negligible. Nevertheless, we sought to explore an alternative approach that is more sensitive than the densitometric measurement of chemically stained HPTLC plates. We analyzed the lipids by reversed-phase HPLC-Q-TOF-MS. In the negative mode, we identified the most abundant species in AcPIM2 ([M-H]^-^ *m/z* 1413.8998) and Ac_2_PIM2 ([M-H]^-^ *m/z* 1652.1281) lipid classes in cells without and with RMS-treatment, respectively (Fig. 7A, B). The relative intensities quantified as chromatogram areas for AcPIM2 versus Ac_2_PIM2 detected were consistent with the results seen by HPTLC analysis. Since lipid classes can differ in MS response factors, we sought to quantitate the amounts of AcPIM2, Ac_2_PIM2, and other major phospholipids in absolute terms in both cellular conditions, using five synthetic standards: CL, PE, PI, PIM2, and Ac_2_PIM2 with serial dilutions to generate external standard curves (Fig. 7C). We listed four major chain length and unsaturation variants in CL, PE, PI, AcPIM2, and Ac_2_PIM2 found in the bacterial samples (Fig. 7D). The lipid quantities for CL, PE, PI, and Ac_2_PIM2 were estimated using the external standard curve fitting. For AcPIM2, no standard was available, so quantities were estimated by curve fitting with assumption that AcPIM2 has an average response factor of two related molecules (Fig. 7E).

All four experiments showed the consistent results, finding that the quantity of Ac_2_PIM2 increased significantly when cells are treated with RMS compared to untreated cells (Fig. 7F). When comparing the molar ratio of Ac_2_PIM2 to AcPIM2, there was a 100-fold increase when cells are treated with RMS compared to untreated cells (Fig. 7G).

Finally, we measured the lipid quantity of CL, PE, and PI and compared these major phospholipids with AcPIM2 and Ac_2_PIM2 in cells treated with and without RMS (Fig. 7H). The quantity of AcPIM2 was substantially lower than the other phospholipids in the cells without RMS treatment, representing only ∼7% of the total phospholipids (Table 1). Furthermore, while the quantity of Ac_2_PIM2 increases when cells are treated with RMS, it remains a relatively minor component of the plasma membrane in comparison to CL, PE, and PI (Fig. 7H). With or without RMS treatment, PIM2 species, both AcPIM2 and Ac_2_PIM2 combined, did not exceed any of the major phospholipids in molar quantity.

**Table 1:** Molar ratio of phospholipids in *M. smegmatis*

Treatment	CL	PE	PI	AcPIM2	Ac_2_PIM2
Without RMS	36.4	32.7	23.6	7.1	0.17
With RMS	43.2	20.9	20.9	3.7	10.6

Ac_2_PIM2, diacyl phosphatidylinositol dimannoside; AcPIM2, monoacyl PI dimannoside; CL, cardiolipin; PE, phosphatidylethanolamine; PI, phosphatidylinositol; RMS, reverse micellar solution.

Shown in % of total major phospholipids.

## Discussion

PIMs are crucial glycolipids found in the mycobacterial plasma membrane. PIM biosynthesis is dependent on inositol, and the inositol auxotroph of *M. smegmatis*, Δ*ino1*, succumbs to inositol starvation. Notably, the timing of mortality does not align with the depletion of PI, which is the most abundant inositol-containing lipid, but rather with the depletion of AcPIM6 and Ac_2_PIM6 (7). The Δ*pimE* strain, deficient in AcPIM6 and accumulating AcPIM4 instead, exhibits heightened susceptibility to antibiotics and increased permeability of its cell envelope to ethidium bromide compared to the wildtype (32). Additionally, electron microscopy reveals plasma membrane deformations in the Δ*pimE* mutant (16). Diacyl PIMs such as Ac_2_PIM2 and Ac_2_PIM6 accumulate under stress conditions (9), with inositol acylation particularly rapid in response to membrane fluidization stress (19). Developing an accurate model of the mycobacterial plasma membrane is crucial for understanding the physiological roles of PIMs at the molecular level. Recent computational modeling studies, following the proposal by Bansal-Mutalik and Nikaido (4), have made an assumption that Ac_2_PIM2 predominates the cytoplasmic leaflet of the plasma membrane (33, 34). These studies suggested that cytoplasmic leaflet was a more dense and less fluid layer with large domains of Ac_2_PIM2. However, our current research suggests that such dominance of Ac_2_PIM2 in the inner leaflet of the plasma membrane is improbable under standard laboratory growth conditions, implying a need for revisiting these modeling studies..

Mycobacteria remain viable when suspended in aliphatic hydrocarbons like petroleum ether (35). Aliphatic hydrocarbons are immiscible with aqueous bacterial cells and therefore do not kill mycobacteria immediately. However, they can extract surface-exposed lipids, and this feature has provided a convenient method to remove surface lipids and redecorate the stripped surface with exogenously supplied lipids (36). Since the RMS is a solution of sodium docusate in heptane, we wondered if the cells remain viable in this aliphatic hydrocarbon solution. In the current study, we demonstrated that *M. smegmatis* not only survives RMS treatment but also responds to sodium docusate, the anionic detergent component of the RMS, resulting in PIM inositol acylation. Given the agreement between HPTLC staining and quantitative MS results, we propose that Ac_2_PIM2 does not dominate the membrane in a resting unperturbed state. This conclusion is supported by previously published results using Molybdenum Blue staining (16, 31) and [^32^P]phosphate metabolic labeling (1, 2). Even after RMS treatment, our MS data indicated that PIMs remain a relatively minor component of the plasma membrane. However, we acknowledge that PIM-dominant plasma membrane may be possible in other species of *Mycobacterium* as reported previously (5, 6, 37). Given the dynamic nature of mycobacterial plasma membrane, it also remains possible that *M. smegmatis* produces a PIM-dominant plasma membrane under nonstandard growth conditions.

We speculate that PIMs are likely accumulating on the periplasmic leaflet of the plasma membrane based on the following observations. First, because both AcPIM2 and AcPIM6 are subject to rapid inositol acylation with similar kinetics (19), we speculate that they are both available on the same leaflet of the plasma membrane. Second, PimE, the fifth mannosyltransferase involved in AcPIM6 synthesis (16), is an integral membrane protein that belongs to the GlycosylTransferase Family 87 within the GT-C structural superfamily in the Carbohydrate Active Enzymes database (http://www.cazy.org/) (38, 39, 40). As a typical GT-C superfamily enzyme, PimE uses polyprenol-phosphate-mannose as the donor of mannose, and its active site is proposed to face the periplasmic side (16, 41). Furthermore, in a cell-free system, the productions of AcPIM4, AcPIM5, and AcPIM6 were inhibited by amphomycin, an inhibitor of polyprenol-phosphate-mannose synthesis (17). Therefore, we consider that AcPIM6 is made in the extracytoplasmic leaflet of the plasma membrane. When considering these two observations, it seems likely that both Ac_2_PIM2 and Ac_2_PIM6 are produced on the periplasmic leaflet of the plasma membrane. We acknowledge that other scenarios are possible. For example, Ac_2_PIM2 produced on the periplasmic side can then be flipped back to the cytoplasmic leaflet. Such a scenario can support the proposal by Bansal-Mutalik and Nikaido that Ac_2_PIM2 is primarily found in the cytoplasmic leaflet of the plasma membrane (4), but there is no evidence in the literature that supports such a scenario.

Mycobacteria produce a diderm cell envelope, and the outer membrane may also contain phospholipids. Bansal-Mutalik and Nikaido indicated that the RMS treatment was ineffective in extracting phospholipids (4), suggesting that the outer membrane lacks major phospholipid species such as CL, PE, and PI. Our data also showed that substantial amounts of phospholipids remain associated with the cell after the RMS treatment, indicating an enrichment of major phospholipid species in the plasma membrane. While we observed that some phospholipids appeared to be extracted by the RMS treatment, the relative ratio of CL, PE, and PI appeared similar with or without prior RMS treatment (see Fig. 6A). These observations imply that phospholipid compositions may be similar between the plasma membrane and the outer membrane. Using a different method of gentle bead beating, an older study indicated that PE and PIMs are released from the cell surface of various *Mycobacterium* species, including *M. smegmatis* (42). The same group partially purified the cell wall–outer membrane fraction in a more recent study and suggested that major phospholipids are found in both the plasma membrane and outer membrane (43). Additional studies are needed to determine precise contributions of major phospholipids to the composition of the outer membrane.

In summary, diacyl forms of PIMs such Ac_2_PIM2 and Ac_2_PIM6 do accumulate in response to stress, but based on available information on the PIM biosynthetic pathway, we suspect that PIMs are present in the extracytoplasmic leaflet. Furthermore, even in the plasma membrane under stress, our data indicate that the dominant structural phospholipids of *M. smegmatis* plasma membrane are PE, CL, and PI.

## Data availability

All data are contained within the manuscript.

## Supplemental data

This article contains supplemental data.

## Conflict of interest

The authors declare that they have no conflicts of interest with the contents of this article.
