# Supplementary material for: Detergent-induced quantitatively limited formation of diacyl phosphatidylinositol dimannoside in Mycobacterium smegmatis
Source: J Lipid Res. 2024 Mar 24;65(7):100533. doi: 10.1016/j.jlr.2024.100533 (PMC11269278; doi:10.1016/j.jlr.2024.100533)
Supplement: Supplemental Figure [file mmc1.pdf]

Supplemental Data

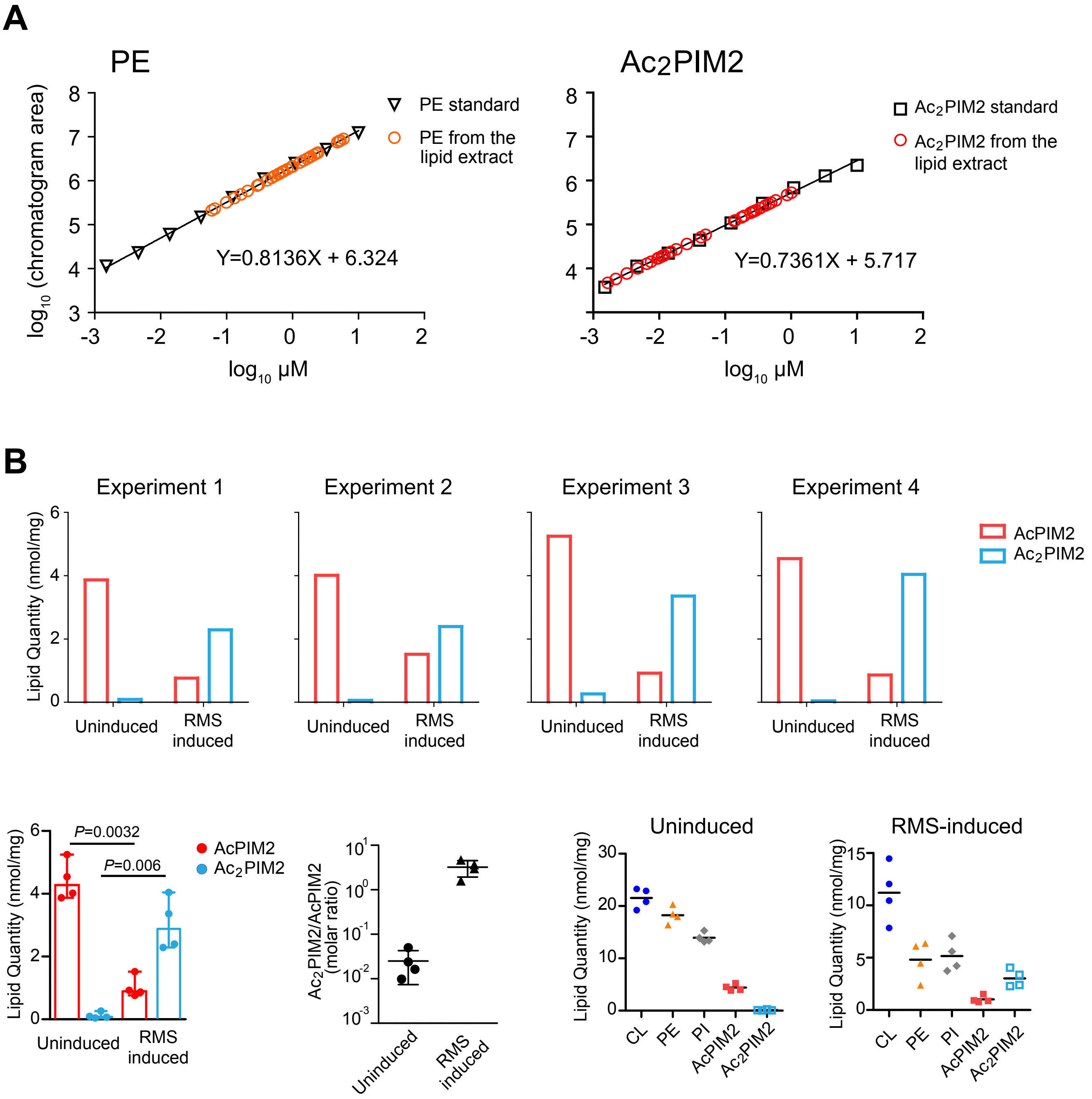

Supplemental Figure S1. Verification of nonlinear standard curve fitting. (**A**) The non-linear standard curves of PE and Ac_2PIM2 in Figure 7C were plotted as logarithmic scale for both x- and y-coordinates. The converted standard curves PE (black triangle) and Ac_2PIM2 (black square) were fitted with linear equations as indicated, which were used to calculate PE (orange circle) or Ac_2PIM2 (red circle) concentrations in the extracted lipids. (**B**) The estimated lipid quantities using the linear curve fitting were comparable with the results obtained by the non-linear curve fitting in Figure 7E-H.
